# PI-QUAL version 2: an update of a standardised scoring system for the assessment of image quality of prostate MRI

**DOI:** 10.1007/s00330-024-10795-4

**Published:** 2024-05-24

**Authors:** Maarten de Rooij, Clare Allen, Jasper J. Twilt, Linda C. P. Thijssen, Patrick Asbach, Tristan Barrett, Giorgio Brembilla, Mark Emberton, Rajan T. Gupta, Masoom A. Haider, Veeru Kasivisvanathan, Vibeke Løgager, Caroline M. Moore, Anwar R. Padhani, Valeria Panebianco, Philippe Puech, Andrei S. Purysko, Raphaële Renard-Penna, Jonathan Richenberg, Georg Salomon, Francesco Sanguedolce, Ivo G. Schoots, Harriet C. Thöny, Baris Turkbey, Geert Villeirs, Jochen Walz, Jelle Barentsz, Francesco Giganti

**Affiliations:** 1 Department of Medical Imaging, Radboud University Medical Center, Nijmegen, The Netherlands; 2 Department of Radiology, University College London Hospital NHS Foundation Trust, London, UK; 3 Department of Radiology, Charité - Universitätsmedizin Berlin, Corporate Member of Freie Universität Berlin, Humboldt-Universität zu Berlin, and Berlin Institute of Health, Berlin, Germany; 4 Department of Radiology, Addenbrooke’s Hospital and University of Cambridge, Cambridge, UK; 5 Department of Radiology, IRCCS San Raffaele Scientific Institute, Vita-Salute San Raffaele University, Milan, Italy; 6 Division of Surgery and Interventional Science, University College London, London, UK; 7 Department of Urology, University College London Hospital NHS Foundation Trust, London, UK; 8 Department of Radiology, Duke University Medical Center, Durham, NC USA; 9 Joint Department of Medical Imaging, Sinai Health System, Lunenfeld Tanenbaum Research Institute, University of Toronto, Toronto, Canada; 10 Department of Radiology, Herlev Gentofte University Hospital, Herlev, Denmark; 11 Paul Strickland Scanner Centre, Mount Vernon Hospital, Northwood, Middlesex UK; 12 Department of Radiological Sciences, Oncology and Pathology, Sapienza University/Policlinico Umberto I, Rome, Italy; 13 Department of Radiology, CHU Lille, University Lille, Lille, France; 14 Abdominal Imaging Section and Nuclear Radiology Department, Diagnostic Institute, and Glickman Urological and Kidney Institute Cleveland Clinic, Cleveland, OH USA; 15 AP-HP, Radiology, Pitié-Salpêtrière Hospital, Sorbonne University, Paris, France; 16 Department of Imaging, Sussex universities Hospitals NHS Foundation Trust, Brighton, UK; 17 Martini Clinic (Prostate Cancer Centre), University of Hamburg, Hamburg, Germany; 18 Department of Medicine, Surgery and Pharmacy, Università degli Studi di Sassari, Sassari, Italy; 19 Department of Urology, Fundació Puigvert, Barcelona, Spain; 20 Department of Radiology & Nuclear Medicine, Erasmus University Medical Centre, Rotterdam, The Netherlands; 21 Department of Radiology, Netherlands Cancer Institute, Amsterdam, The Netherlands; 22 Department of Diagnostic and Interventional Radiology, Fribourg Cantonal Hospital, Fribourg, Switzerland; 23 Molecular Imaging Branch, National Cancer Institute, National Institutes of Health, Bethesda, MD USA; 24 Department of Medical Imaging, Ghent University Hospital, Ghent, Belgium; 25 Department of Urology, Institut Paoli-Calmettes Cancer Centre, Marseille, France; 26 Andros Clinics, Arnhem, The Netherlands

**Keywords:** Magnetic resonance imaging, Prostatic neoplasms, Quality control

## Abstract

**Abstract:**

Multiparametric MRI is the optimal primary investigation when prostate cancer is suspected, and its ability to rule in and rule out clinically significant disease relies on high-quality anatomical and functional images.

Avenues for achieving consistent high-quality acquisitions include meticulous patient preparation, scanner setup, optimised pulse sequences, personnel training, and artificial intelligence systems. The impact of these interventions on the final images needs to be quantified.

The prostate imaging quality (PI-QUAL) scoring system was the first standardised quantification method that demonstrated the potential for clinical benefit by relating image quality to cancer detection ability by MRI.

We present the updated version of PI-QUAL (PI-QUAL v2) which applies to prostate MRI performed with or without intravenous contrast medium using a simplified 3-point scale focused on critical technical and qualitative image parameters.

**Clinical relevance statement:**

High image quality is crucial for prostate MRI, and the updated version of the PI-QUAL score (PI-QUAL v2) aims to address the limitations of version 1. It is now applicable to both multiparametric MRI and MRI without intravenous contrast medium.

**Key Points:**

- *High-quality images are essential for prostate cancer diagnosis and management using MRI*.
- *PI-QUAL v2 simplifies image assessment and expands its applicability to prostate MRI without contrast medium*.
- *PI-QUAL v2 focuses on critical technical and qualitative image parameters and emphasises T2-WI and DWI*.

## Introduction

The introduction of multiparametric MRI (mpMRI) has led to a paradigm shift in the diagnostic pathway for prostate cancer. By incorporating prostate MRI before biopsy, overdiagnosis and overtreatment of indolent cancers have been reduced due to a decrease in unnecessary biopsies [1–4]. In addition, MRI-guided biopsies detect at least as many clinically significant prostate cancers as traditional approaches using systematic transrectal ultrasound-guided biopsies. Consequently, the integration of MRI into prostate cancer guidelines has led to a substantial increase in routine use [5]. Multiple other applications of prostate MRI have emerged including local staging, population screening and the active surveillance of patients with indolent disease.

High-quality MR images are a prerequisite for any MRI-driven prostate cancer diagnostic pathway, directly influencing the accuracy of cancer detection and subsequent management decisions [6–8]. Despite the implementation of the Prostate Imaging Reporting and Data System (PI-RADS) version 2.1 technical standards, wide variability in image quality persists in community practice [9–11]. This variation is not only caused by poor adherence to the PI-RADS technical requirements, but is also influenced by factors such as patient motion, metalwork, patient preparation, and scanner hardware including receiver coils, scanner performance, software level and field strength [12–14].

Consequently, there is a growing recognition among urological and radiological societies of the need for an easy-to-use tool to objectively assess the quality of prostate MRI images [15, 16]. The first attempt to standardise the assessment of image quality was the prostate imaging quality (PI-QUAL) scoring system [17] developed by researchers of the PRECISION trial [18]. The first version of PI-QUAL (PI-QUAL v1) categorises image quality on a 5-point scale by evaluating each MRI sequence against a defined set of technical criteria along with subjective assessments of image quality for each multiparametric sequence [T2-weighted imaging (T2-WI), diffusion-weighted imaging (DWI), and dynamic contrast-enhanced (DCE) MRI].

PI-QUAL v1 has been evaluated in different patient cohorts and it has been demonstrated that image quality has a direct bearing on cancer detection and biopsy planning [14]. However, PI-QUAL v1 has limitations [19, 20]; the most important being that it is suitable only for mpMRI examinations, thus excluding examinations without intravenous contrast medium. Furthermore, it does not enable an objective evaluation of image quality distinct from biopsy implications, restricting its use in other clinical scenarios (tumour staging, active surveillance, population screening and follow-up of patients with prior negative/positive scans).

This document sets out the updated version of PI-QUAL (v2). It has been developed by an extended European Society of Urogenital Radiology (ESUR) prostate cancer working group to overcome the limitations mentioned above by (i) accommodating MRI without intravenous contrast medium; (ii) simplifying the scoring process and (iii) ensuring that the scoring system provides a reproducible assessment of the image quality, applicable to a wider range of patients in the clinical routine.

By introducing PI-QUAL v2, we aim to further enhance the standardisation and reliability of prostate MRI quality assessment, thereby optimising the diagnostic accuracy and subsequent management of patients with known or suspected prostate cancer.

## Prostate MR image quality

High image quality is a prerequisite for any MRI-based diagnostic pathway. Suboptimal image quality can result in under or overcalling lesions, inadequate characterisation and staging, or the inability to confidently call negative scans [14, 21]. Poor image quality has been associated with increased rates of indeterminate MRI findings (i.e. PI-RADS 3 lesions) [22]. Thus, image quality can influence subsequent steps in the diagnostic pathway, such as MR-targeted biopsies, risk stratification, and treatment decisions. Prostate MR image quality depends on several aspects, including adherence to the technical standards defined within the PI-RADS guidelines, sufficiently high signal-to-noise ratio (SNR), image contrast, and image sharpness to distinguish and delineate relevant structures in and around the prostate gland.

### Technical recommendations

The technical recommendations outlined in PI-RADS v2.1 [9] encompass critical aspects, including the field strength and basic image acquisition parameters, such as which and how many *b* values should be used for DWI, and the temporal resolution of DCE sequences. Furthermore, the use of endorectal coils is discouraged and phased-array surface coils are preferred for signal reception. These recommendations are specified to harmonise prostate MRI practice across institutions and enhance the reproducibility of MRI for detecting and characterising prostate lesions. An overview of the technical recommendations of PI-RADS v2.1 is presented in Table 1.

**Table 1:** PI-RADS v2.1 technical recommendations

	T2-weighted imaging (T2-WI)	Diffusion-weighted imaging (DWI)	Dynamic contrast-enhancement (DCE)
Imaging planes	Axial (either straight axial to the patient or in an oblique axial plane matching the long axis of the prostate) and at least one additional orthogonal plane (i.e. sagittal and/or coronal)	Imaging planes should match or be similar to those used for T2W and DCE	Imaging planes should match or be similar to those used for T2W and DWI
Slice thickness and interslice gap	3 mm, No gap	≤ 4 mm, No gap	3 mm, No gap
Field of view	12–20 cm*	16–22 cm	12–20 cm*
In-plane dimension	≤ 0.7 mm (Phase) × ≤ 0.4 mm (frequency)	≤ 2.5 mm (Phase and frequency)	≤ 2 mm (Phase and frequency)
Specific recommendations			
	3D axial as an adjunct to 2D acquisitions	–	3D sequences to improve SNR
Low *b* value	–	0 (Preferably 50)–100 s/mm^2^	–
Intermediate *b* value	–	800–1000 s/mm^2^	–
High *b* value	–	- Dedicated (≥ 1400 s/mm^2^) - Synthesised (from other *b* values)	–
ADC		Mono-exponential fitting of *b* values < 1000 s/mm^2^	
Temporal resolution	–	–	≤ 15 s
Total observation rate	–	–	> 2 min
Dose of GBCA	–	–	0.1 mmol/kg Body weight
Injection rate	–	–	2–3 mL/s
Fat suppression/subtraction	–	Mandatory	Recommended

*ADC* apparent diffusion coefficient, *DCE-MRI* dynamic contrast-enhanced MRI, *DWI* diffusion-weighted imaging, *GBCA* Gadolinium-based contrast agent, *T2-WI* T2-weighted imaging

* To encompass the entire prostate gland and seminal vesicles

### Prostate MRI artefacts

Despite the PI-RADS technical recommendations, prostate MR quality remains variable and can be influenced by various factors [14]. Prostate MRI is susceptible to a variety of artefacts including:

1. Motion artefacts, which are among the most prevalent. Given the location of the prostate in the pelvis, involuntary motion, such as respiratory, peristalsis, bladder filling and pelvic floor movements can lead to image blurring.
2. Susceptibility artefacts on DWI are another common concern and are primarily attributed to the presence of diverse tissue types around the prostate with differing magnetic susceptibilities. Challenges arise from the presence of rectal gas or the presence of metals, such as hip prostheses. These artefacts manifest as signal distortions and loss, particularly at the recto–prostatic interface, where a gas-filled rectum can cause inhomogeneities of the magnetic field and geometric distortions of the prostate gland.
3. Chemical shift artefacts can also be observed because of the distinct resonant frequencies of fat and water protons. This phenomenon can lead to the appearance of dark or bright bands at the fat–water interface, potentially obscuring the boundaries of the prostate gland.
4. Aliasing or wrap-around artefacts occur when the field of view is too small for the imaged anatomy. Wrap-around occurs when structures appear at unexpected locations within the image. Adjusting the field of view or using larger matrices can help to reduce these artefacts.
5. Finally, poor SNR is problematic when surface coils are used for signal reception in patients with high body mass index, specifically with large hip circumferences.

### Key factors in the optimisation of image quality

Effectively managing common artefacts is crucial for optimising image quality. The key factors for image quality optimisation can be categorised into three different perspectives.

## Patient preparation

Several patient-centred measures can enhance image quality, but there is no consensus on the optimal strategy for patient preparation. To reduce involuntary motion artefacts, anti-peristaltic agents (e.g. anticholinergic agents, glucagon) can be used, aiming to reduce small bowel and rectal movements. These agents typically have an immediate antiperistalsis effect lasting approximately 20–30 min. The downsides of anti-peristaltic agents include adverse effects, rebound, and additional costs. Moreover, not all patients can receive these medications due to contraindications (e.g. glaucoma in the case of anticholinergic agents) or regulatory limitations. While these agents have been shown to improve the quality [23–25], they have not demonstrated benefits in cancer detection [24] or staging [26]. Other techniques include the use of micro-enemas, dietary restrictions, and thin rectal catheters to relieve rectal air build-up [27]. Due to conflicting results and low levels of scientific evidence on the effectiveness of these measures, along with the lack of diagnostic impacts on prostate cancer diagnosis, centres are urged to investigate and report the local success of different patient preparation methods to help define best practices.

## Hardware

The topics that have been most widely studied are the magnetic field strength (1.5 T vs 3 T) and the use of an endorectal coil. In theory, there is a benefit of using a higher magnetic field strength because of the higher SNR, but the disadvantage to this is a higher risk of susceptibility artefacts. Limited evidence from small studies shows higher image quality from 3 T scanners compared with 1.5 T. However, there is a consensus that adequate image quality is still possible at at 1.5 T with optimised protocols [14]. The PI-RADS v2.1 guidelines recommend the use of 3 T, except in cases of metallic implants or devices [9]. Endorectal coils can be used to increase the SNR mainly for 1.5 T scanners and may also be beneficial for patients with large body mass index [28]. A meta-analysis revealed that the use of an endorectal coil did not show benefits for detecting extra-prostatic extension, and only demonstrated marginal improvement in sensitivity for seminal vesicle invasion [29]. The disadvantages of patient discomfort, additional time, and costs must be balanced with the potential benefits of using an endorectal coil.

## Image acquisition team

It is crucial to recognise that good image quality can only be achieved through teamwork. Centres with state-of-the-art MR scanners, specialist radiographers or radiology technologists, and genitourinary radiologists should, when working together, produce consistently high-quality MR images. The ESUR and the European Association of Urology Section of Urologic Imaging (ESUI) have published recommendations regarding prerequisites for reporting, experience levels, supervision of prostate MRI reporting radiologists, and training of radiographers/technologists [15]. Radiologists and radiographers or radiology technologists are advised to participate in training programs that include prostate MRI image quality as part of the quality assurance processes of diagnostic centres. Enhancing awareness of optimal image quality among radiologists and radiographers necessitates the implementation of standardised and objective metrics, enabling them to impartially assess scans with a critical perspective. Another recommendation is that image quality should be recorded in routine clinical reporting for clinical audits and quality control. These recommendations can serve as a starting point for quality-assuring a prostate imaging pathway.

## PI-QUAL version 2

### Methodology

PI-QUAL v2 is a result of a collaborative international effort that engaged 20 experienced genitourinary radiologists specialising in prostate MRI, along with a group of six urologists actively incorporating prostate MRI into their routine clinical practice. This initiative brought together experts from Europe and North America who were working members of the Prostate Subgroup of the ESUR and ESUI and selected invited members from the Society of Abdominal Radiology (SAR) prostate cancer disease focused panel. Note that this work product is not endorsed by the SAR with members contributing in their individual capacities.

The working group was chaired by two radiologists (M.d.R. and F.G.) who facilitated the discussions through virtual and in-person meetings, as well as email correspondence between June 2021 and February 2024. Initial drafts were refined stepwise through testing on multiple cases to improve objectivity and increase reliability.

To gauge the initial inter-reader agreement of the scoring system, six out of the 20 radiologists who were not part of the initial development of PI-QUAL v2, independently evaluated 50 studies with varying image quality. These studies comprised 25 MRI scans without contrast medium and 25 mpMRI scans obtained from different vendors and magnets, selected randomly by the two chairs. The inter-reader agreement, calculated using the percentage of agreement with linear weighting, was 61%.

All authors involved in this study played an active role in defining, refining and editing PI-QUAL v2.

### PI-QUAL v2 scoring system

All centres conducting prostate MRIs should be aware of and largely comply with the technical recommendations outlined in the PI-RADS v2.1 guidelines [9] (Table 1). However, certain PI-RADS technical parameters carry greater importance for obtaining images with optimal diagnostic capabilities. Adjustments in certain parameters such as field of view and in-plane resolution can help improve SNR [30]. Therefore, it was unanimously agreed that the new version of PI-QUAL should include only critical technical prerequisites for each sequence before proceeding with image quality assessments (Table 2).

**Table 2:** Essential technical prerequisites per sequence

T2-WI	DWI	DCE
3 mm Slice thickness	≤ 4 mm Slice thickness	3 mm Slice thickness
	High *b* value sequence (≥ 1400 s/mm^2^), calculated or acquired	Temporal resolution ≤ 15 s
	ADC map using at least two *b* values up to 1000 s/mm^2^	Fat suppression (or include post-processing, e.g. subtraction/heat maps) 3D sequences (preferred)

*T2-WI* T2-weighted imaging, *DWI* diffusion-weighted imaging, *DCE* dynamic contrast-enhancement, *ADC* apparent diffusion coefficient

**Fig. 1:**
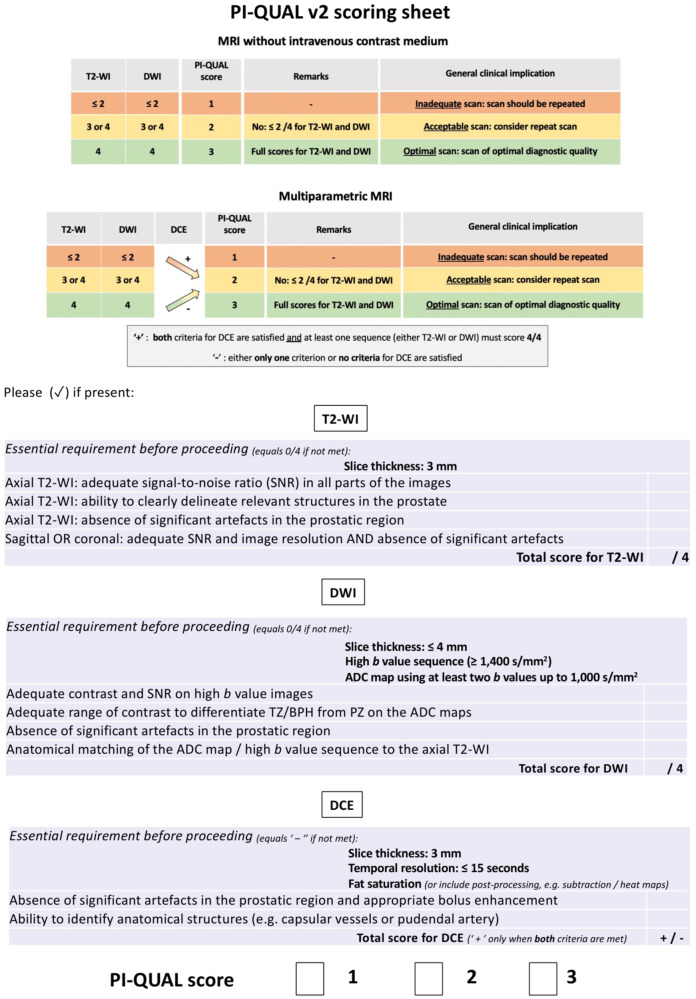
PI-QUAL v2 scoring sheet that includes the basic mandatory PI-RADS v2.1 technical prerequisites for T2-WI, DWI and DCE, and the table to derive the PI-QUAL v2 score

The scoring sheet for PI-QUAL v2 comprises 10 criteria that include the ability to clearly delineate the relevant structures in the prostate (e.g. the capsule, seminal vesicles, ejaculatory ducts, neurovascular bundles, and external urethral sphincter) and the assessment of the most prevalent artefacts and image degradations that severely affect the prostate for each sequence individually (Fig. 1).

**Fig. 2:**
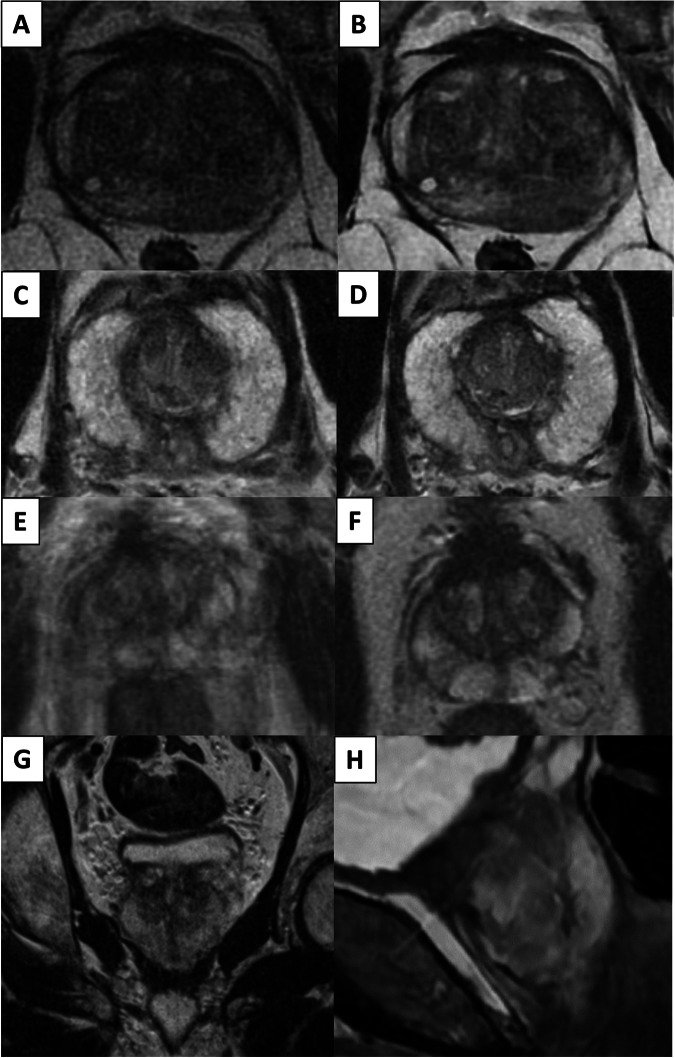
The four T2-WI criteria: examples with degraded axial (**A**, **C**, and **E**), coronal (**G**) and sagittal (**H**) T2-WI. Axial T2-WI of optimal image quality (**B**, **D**, and **F**). Axial T2-weighted images are from the same patients (before and after correction)
- Four criteria for DWI (maximum score: 4/4) (Fig. 3)

There are:

- Four criteria for T2-WI (maximum score: 4/4) (Fig. 2)

**Fig. 3:**
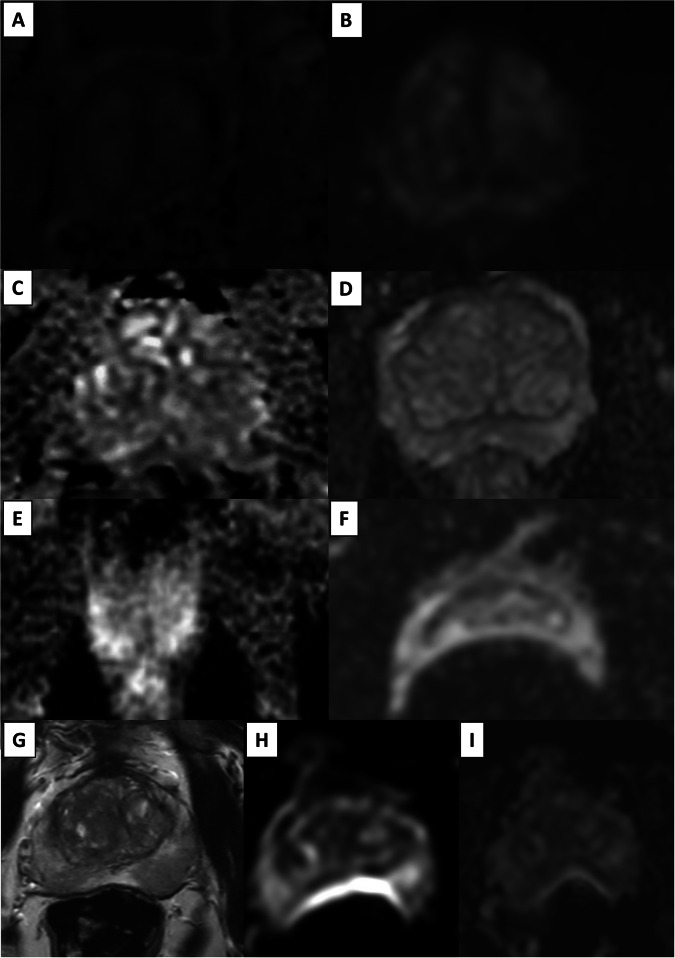
The four DWI criteria: examples with degraded (**A**, **C**, **E**, **F**, **H** and **I**) and optimal (**B**, **D**, and **G**—this latter is axial T2-WI) image quality. Significant susceptibility artefacts can cause displacement/distortion, which is regarded as severe when the displacement is > 5 mm versus axial T2-WI (**G**) at the posterior surface of the prostate (**H**–**I**)
- Two criteria for DCE sequences (dichotomised score: ‘+/−’) (Fig. 4).

**Fig. 4:**
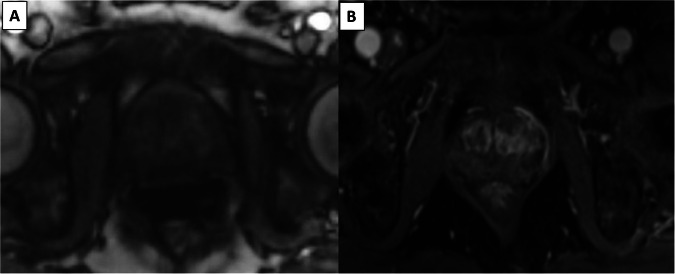
The two DCE criteria: examples with degraded (**A**) and optimal (**B**) image quality

In keeping with the PI-RADS v2.1 recommendations, T2-WI and DWI are the dominant sequences and can score up to four quality points each, while there are only two separate criteria for DCE sequences (and only when both criteria are met, are DCE sequences considered of optimal diagnostic quality). This aligns with the lesser role of DCE in the PI-RADS v2.1 scoring system, where it is primarily used to upgrade PI-RADS 3 to PI-RADS 4 lesions in the peripheral zone, with no role in the transition zone for category assignment of detected lesions.

The new PI-QUAL score is shown in Fig. 1 and the rules used to derive the PI-QUAL score for MRI without intravenous contrast medium and for mpMRI are shown in Table 3.

**Table 3:** Flowchart to assess the PI-QUAL score using MRI without or with intravenous contrast medium

T2-WI	DWI	PI-QUAL (no contrast)	DCE	PI-QUAL (mpMRI)	Comparison
1 or 2	1 or 2	1	−	1	=
3	1 or 2	1	−	1	=
4	1 or 2	1	−	1	=
1 or 2	3	1	−	1	=
3	3	2	−	2	=
4	3	2	−	2	=
1 or 2	4	1	−	1	=
3	4	2	−	2	=
4	4	3	−	2	Downgrade
1 or 2	1 or 2	1	+	1	=
3	1 or 2	1	+	1	=
4	1 or 2	1	+	2	Upgrade
1 or 2	3	1	+	1	=
3	3	2	+	2	=
4	3	2	+	2	=
1 or 2	4	1	+	2	Upgrade
3	4	2	+	2	=
4	4	3	+	3	=

*T2-WI* T2-weighted imaging, *DWI* diffusion-weighted imaging, *PI-QUAL* prostate imaging quality, *DCE* dynamic contrast-enhanced, *mpMRI* multiparametric magnetic resonance imaging

Summary quality categories for MRI without intravenous contrast medium:

- PI-QUAL score of 1: the image quality is inadequate (i.e. T2-WI and/or DWI score ≤ 2/4). The examination does not meet the critical technical/image quality requirements.
- PI-QUAL score of 2: the image quality is acceptable (i.e. T2-WI and DWI score at least 3/4).
- PI-QUAL score of 3: the image quality is optimal (i.e. T2-WI and DWI both score 4/4).

Summary Quality Categories for mpMRI:

- PI-QUAL score of 1: the image quality is inadequate (i.e. T2-WI and/or DWI score ≤ 2/4 and either only one criterion or no criteria for DCE sequences are satisfied). The examination does not meet the critical technical/image quality requirements. However, if both criteria for DCE sequences are satisfied and at least one sequence (either T2-WI or DWI) scores 4/4, the PI-QUAL score is upgraded to PI-QUAL 2.
- PI-QUAL score of 2: the image quality is acceptable (i.e. T2-WI and DWI score at least 3/4). This score cannot be upgraded or downgraded by DCE sequences.
- PI-QUAL score of 3: the image quality is optimal (i.e. T2-WI and DWI both score 4/4 and both criteria for DCE sequences are satisfied). However, if only one criterion or no criteria for DCE sequences are met, the PI-QUAL score is downgraded to PI-QUAL 2.

It is important to focus on the differences between the two scanning protocols (with or without intravenous contrast medium).

Upgrading or downgrading of the PI-QUAL score can occur in three scenarios when using mpMRI:

- Scenario 1: full scores (i.e. 4/4) both for T2-WI and DWI, but DCE of suboptimal quality.

The PI-QUAL score would be 3 for MRI without intravenous contrast medium but PI-QUAL 2 on mpMRI. Note that a PI-QUAL score of 2 is also given when either T2-WI or DWI is not optimal (i.e. not 4/4) for MRI without intravenous contrast medium.

- Scenario 2: full scores (i.e. 4/4) for T2-WI, but not for DWI (i.e. 1 or 2 out of 4), but DCE of optimal diagnostic quality.

The PI-QUAL score would be 1 for MRI without intravenous contrast medium but PI-QUAL 2 on mpMRI. This would occur in the presence of pelvic metalwork. In this scenario, optimal DCE sequences represent a ‘safety net’.

- Scenario 3: full scores (i.e. 4/4) for DWI, but not for T2-WI (i.e. 1 or 2 out of 4), but DCE of optimal diagnostic quality.

The PI-QUAL score would be 1 for MRI without intravenous contrast medium but PI-QUAL 2 on mpMRI. Again, optimal DCE sequences represent a ‘safety net’.

Further remarks:

i. The panel agreed that the all-prostate gland relevant acquired images be evaluated together for their net contribution to PI-QUAL image quality. Thus, mpMRI quality must be evaluated with DCE images for an overall quality assessment. Selective reassignments of image quality based on the quality of DCE images is strongly discouraged.
ii. There is no overall sum score. For example, a score of 5/8 for MRI without intravenous contrast medium is not advised. This implies that if either T2-WI or DWI scores ≤ 2/4, the PI-QUAL score for an MRI without intravenous contrast medium is automatically 1 (i.e. scan of inadequate diagnostic quality) irrespective of the possibility that the other sequence (either T2-WI or DWI) may still achieve a higher score.
iii. It is recommended to include the separate score of each sequence along with the final PI-QUAL v2 score, to gain insight into which sequence needs to be improved. Describing why a particular sequence is limited is advisable, for instance: *“*Image quality assessment: T2-WI: 3/4; DWI: 2/4 and both DCE criteria met (i.e. ‘+’), resulting in a final PI-QUAL score of 1*”*. This indicates inadequate image quality attributed to an insufficient T2-WI and DWI sequence, resulting for instance from minor motion artefacts on T2-WI, the lack of discrimination of the peripheral zone from the transition zone on DWI and significant susceptibility artefacts on the apparent diffusion coefficient (ADC) image due to rectal air.
iv. Ideally, poor-quality sequences should be repeated while the patient is undergoing the examination, and the pre-contrast T1 sequences should be checked for the absence of artefacts before intravenous injections.
v. The differences between PI-QUAL v1 and PI-QUAL v2 are shown in Table 4.

**Table 4:** Major differences between PI-QUAL v1 and PI-QUAL v2

PI-QUAL v1 (2020)	PI-QUAL v2 (2024)
1–5 Scale	1–3 Scale
Only mpMRI	Both mpMRI and MRI without intravenous contrast medium
Developed by researchers of the PRECISION trial	Developed by an expanded international working group
34 Criteria	10 Criteria
Evaluates compliance with all PI-RADS v. 2 technical recommendations	Defines essential technical requirements based on PI-RADS v2.1 minimum technical requirements for each sequence before assessment
All sequences have the same weighting	T2-WI and DWI have more weighting than DCE sequences

*mpMRI* multiparametric magnetic resonance imaging, *PI-RADS* prostate Imaging reporting and data system, *DCE* dynamic contrast-enhanced, *DWI* diffusion-weighted imaging, *T2-WI* T2-weighted imaging

### Clinical recommendations

PI-QUAL v2 describes the quality of the MR images and should be used to guide clinical decisions about whether it is necessary to repeat an examination. That is, the PI-QUAL score should inform, but not determine, clinical decision-making. For example, in some cases it is still possible to identify a large lesion in an examination of inadequate diagnostic quality (PI-QUAL score 1), allowing a targeted biopsy to be performed without delay, but in such cases, inaccurate staging can still occur thereby affecting the treatment pathway.

It is important to stress that when the diagnostic quality of a scan is inadequate, the PI-RADS or Likert cancer likelihood scores should not be given. Specifically, it is suggested that an inadequate quality scan should NOT be allocated a PI-RADS/LIKERT score of 3. In case of a scan with inadequate quality, the imaging team should investigate and aim to remedy the cause(s). If inadequate diagnostic quality stems from patient-related factors (e.g. movement), while the scanner parameters are satisfactory, measures should be implemented to alleviate this issue. If the inadequate diagnostic quality stems from machine-related factors, the patient should be scanned using another MR system with better performance.

Optimal diagnostic quality (PI-QUAL score 3) is of particular importance when assessing patients on active surveillance or after treatment, where it is crucial to rule in and rule out the presence of clinically significant disease to assess the degree of radiological change over time or the presence of residual/recurrent disease with high confidence.

It should be also kept in mind that a PI-QUAL score of 2 does not mean that rescanning is always needed. Only if the scan remains doubtful or deemed of insufficient quality to make a diagnosis, rescanning is highly advised.

## Future directions

PI-QUAL v2 is a tool for assessing imaging quality, and testing its effectiveness in both research and clinical practice in diverse clinical settings is strongly encouraged. One of the key aspects of the successful adoption of PI-QUAL v2 is teaching its application to those involved in prostate MRI acquisition and reporting. Training on image evaluations by PI-QUAL can be done effectively by dedicated teaching including hands-on sessions [31, 32]. We advocate that dedicated hands-on courses on image quality should become the cornerstone for the successful delivery of the MRI diagnostic pathway for radiologists and radiographers, as well as for radiology trainees and urologists [26].

Image quality assessment should also be a crucial aspect of prostate MRI reports and prostate cancer clinical research. Future and ongoing studies should always report on image quality as part of the scientific reports. Diagnostic centre accreditations should also incorporate PI-QUAL assessments as part of quality assurance and control processes.

Although PIQUAL v2 is designed to provide an objective way to assess image quality, users’ experience and the inherently subjective nature of image quality may lead to some inter- and intra-reader variability. Understanding the extent of this variability will be crucial. For an effective scoring system, intra-reader variability should be less than inter-reader variability and further studies evaluating this aspect are encouraged.

Automated methods based on deep learning have the potential to provide a more reproducible and standardised assessment of image quality. The collection of cases where image quality is routinely annotated would be helpful for model training. Preliminary studies of automated systems utilising convolutional neural networks have undergone testing and demonstrated their capability to accurately identify low-quality prostate MR images [33, 34]. Artificial intelligence assessments of image quality during the scanning process with timely corrective measures could enhance the workflow and overall quality of the MRI diagnostic pathway.

## Conclusions

PI-QUAL v2 for assessing image quality of prostate MRI rectifies the limitations of PI-QUAL v1. The updated scoring system has been simplified by focusing on assessing compliance with critical technical and image quality parameters. Additionally, it applies both to mpMRI and MRI without intravenous contrast medium. This new version, through education, has the potential for broad adoption. As a living document, refinements based on future research and experience in clinical practice are welcome.

Our aspiration is that PI-QUAL v2 will be a key tool in the global effort to improve prostate MR image quality and thus the clinical utility of prostate MRI.

## Abbreviations

mpMRI: Multiparametric MRI
PI-QUAL: Prostate imaging quality
PI-QUAL v2: Updated version of PI-QUAL
SNR: Signal-to-noise ratio

## References

[1] van der Leest M, Cornel E, Israel B et al (2019) Head-to-head comparison of transrectal ultrasound-guided prostate biopsy versus multiparametric prostate resonance imaging with subsequent magnetic resonance-guided biopsy in biopsy-naive men with elevated prostate-specific antigen: a large prospective multicenter clinical study. Eur Urol 75:570–578 30477981 10.1016/j.eururo.2018.11.023

[2] Rouvière O, Puech P, Renard-Penna R et al (2019) Use of prostate systematic and targeted biopsy on the basis of multiparametric MRI in biopsy-naive patients (MRI-FIRST): a prospective, multicentre, paired diagnostic study. Lancet Oncol 20:100–109 30470502 10.1016/S1470-2045(18)30569-2

[3] Kasivisvanathan V, Stabile A, Neves JB et al (2019) Magnetic resonance imaging-targeted biopsy versus systematic biopsy in the detection of prostate cancer: a systematic review and meta-analysis. Eur Urol 76:284–303 10.1016/j.eururo.2019.04.043 31130434

[4] Ahmed HU, El-Shater Bosaily A, Brown LC et al (2017) Diagnostic accuracy of multi-parametric MRI and TRUS biopsy in prostate cancer (PROMIS): a paired validating confirmatory study. Lancet 389:815–822 28110982 10.1016/S0140-6736(16)32401-1

[5] Mottet N, van den Bergh RCN, Briers E et al (2021) EAU-EANM-ESTRO-ESUR-SIOG guidelines on prostate cancer-2020 update. Part 1: screening, diagnosis, and local treatment with curative intent. Eur Urol 79:243–262 33172724 10.1016/j.eururo.2020.09.042

[6] Barrett T, de Rooji M, Giganti F, Allen C, Barentsz JO, Padhani AR (2023) Quality checkpoints in the MRI-directed prostate cancer diagnostic pathway. Nat Rev Urol 20:9–22 36168056 10.1038/s41585-022-00648-4

[7] Padhani AR, Schoots IG, Turkbey B, Giannarini G, Barentsz JO (2021) A multifaceted approach to quality in the MRI-directed biopsy pathway for prostate cancer diagnosis. Eur Radiol 31:4386–4389 33241520 10.1007/s00330-020-07527-9

[8] Dinneen E, Allen C, Strange T et al (2022) Negative mpMRI rules out extra-prostatic extension in prostate cancer before robot-assisted radical prostatectomy. Diagnostics (Basel) 12:1057 10.3390/diagnostics12051057 PMC9139507 35626214

[9] Turkbey B, Rosenkrantz AB, Haider MA et al (2019) Prostate imaging reporting and data system version 2.1: 2019 update of prostate imaging reporting and data system version 2. Eur Urol 76:340–351 30898406 10.1016/j.eururo.2019.02.033

[10] Sackett J, Shih JH, Reese SE et al (2020) Quality of prostate MRI: Is the PI-RADS standard sufficient? Acad Radiol 28:199–207 10.1016/j.acra.2020.01.031 PMC8459209 32143993

[11] Giganti F, Ng A, Asif A et al (2023) Global variation in magnetic resonance imaging quality of the prostate. Radiology 309:e231130 37815448 10.1148/radiol.231130

[12] Burn PR, Freeman SJ, Andreou A, Burns-Cox N, Persad R, Barrett T (2019) A multicentre assessment of prostate MRI quality and compliance with UK and international standards. Clin Radiol 74:894.e19–894.e25 10.1016/j.crad.2019.03.026 31296337

[13] Lin Y, Yilmaz EC, Belue MJ, Turkbey B (2023) Prostate MRI and image quality: it is time to take stock. Eur J Radiol 161:110757 36870241 10.1016/j.ejrad.2023.110757 PMC10493032

[14] Woernle A, Englman C, Dickinson L et al (2023) Picture perfect: the status of image quality in prostate MRI. J Magn Reson Imaging. 10.1002/jmri.29025 10.1002/jmri.29025 37804007

[15] de Rooij M, Israël B, Tummers M et al (2020) ESUR/ESUI consensus statements on multi-parametric MRI for the detection of clinically significant prostate cancer: quality requirements for image acquisition, interpretation and radiologists’ training. Eur Radiol 30:5404–5416 32424596 10.1007/s00330-020-06929-z PMC7476997

[16] Purysko AS, Tempany C, Macura KJ et al (2023) American College of Radiology initiatives on prostate magnetic resonance imaging quality. Eur J Radiol 165:110937 37352683 10.1016/j.ejrad.2023.110937 PMC10461171

[17] Giganti F, Allen C, Emberton M et al (2020) Prostate imaging quality (PI-QUAL): a new quality control scoring system for multiparametric magnetic resonance imaging of the prostate from the PRECISION trial. Eur Urol Oncol 3:615–619 32646850 10.1016/j.euo.2020.06.007

[18] Kasivisvanathan V, Rannikko AS, Borghi M et al (2018) MRI-targeted or standard biopsy for prostate-cancer diagnosis. N Engl J Med 378:1767–1777 29552975 10.1056/NEJMoa1801993 PMC9084630

[19] Barrett T, Lee KL, de Rooij M, Giganti F (2024) Update on optimization of prostate MR imaging technique and image quality. Radiol Clin North Am 62:1–15 37973236 10.1016/j.rcl.2023.06.006

[20] de Rooij M, Barentsz JO (2022) PI-QUAL v.1: the first step towards good-quality prostate MRI. Eur Radiol 32:876–878 34842957 10.1007/s00330-021-08399-3 PMC8628276

[21] Forookhi A, Laschena L, Pecoraro M et al (2023) Bridging the experience gap in prostate multiparametric magnetic resonance imaging using artificial intelligence: a prospective multi-reader comparison study on inter-reader agreement in PI-RADS v2.1, image quality and reporting time between novice and expert readers. Eur J Radiol 161:110749 36812699 10.1016/j.ejrad.2023.110749

[22] Karanasios E, Caglic I, Zawaideh JP, Barrett T (2022) Prostate MRI quality: clinical impact of the PI-QUAL score in prostate cancer diagnostic work-up. Br J Radiol 95:20211372 35179971 10.1259/bjr.20211372 PMC10993954

[23] Slough RA, Caglic I, Hansen NL, Patterson AJ, Barrett T (2018) Effect of hyoscine butylbromide on prostate multiparametric MRI anatomical and functional image quality. Clin Radiol 73:216.e9–216.e14 28803622 10.1016/j.crad.2017.07.013

[24] Sundaram KM, Rosenberg J, Syed AB, Chang ST, Loening AM (2023) Assessment of T2-weighted image quality at prostate MRI in patients with and those without intramuscular injection of glucagon. Radiol Imaging Cancer 5:e220070 37171269 10.1148/rycan.220070 PMC10240243

[25] Ullrich T, Quentin M, Schmaltz AK et al (2018) Hyoscine butylbromide significantly decreases motion artefacts and allows better delineation of anatomic structures in mp-MRI of the prostate. Eur Radiol 28:17–23 28687912 10.1007/s00330-017-4940-7

[26] Engelbrecht MR, Jager GJ, Laheij RJ, Verbeek AL, van Lier HJ, Barentsz JO (2002) Local staging of prostate cancer using magnetic resonance imaging: a meta-analysis. Eur Radiol 12:2294–2302 12195484 10.1007/s00330-002-1389-z

[27] Prabhakar S, Schieda N (2023) Patient preparation for prostate MRI: a scoping review. Eur J Radiol 162:110758 36905717 10.1016/j.ejrad.2023.110758

[28] Robertson SH, Owenby E, Beasley C et al (2023) Optimization of non-endorectal prostate MR image quality using PI-QUAL: a multidisciplinary team approach. Eur J Radiol 166:110998 37506475 10.1016/j.ejrad.2023.110998

[29] de Rooij M, Hamoen EH, Witjes JA, Barentsz JO, Rovers MM (2016) Accuracy of magnetic resonance imaging for local staging of prostate cancer: a diagnostic meta-analysis. Eur Urol 70:233–245 26215604 10.1016/j.eururo.2015.07.029

[30] Abreu-Gomez J, Shabana W, McInnes MDF, O’Sullivan JP, Morash C, Schieda N (2019) Regional standardization of prostate multiparametric MRI performance and reporting: Is there a role for a director of prostate imaging? AJR Am J Roentgenol 213:844–850 31180739 10.2214/AJR.19.21111

[31] Giganti F, Cole AP, Fennessy FM et al (2023) Promoting the use of the PI-QUAL score for prostate MRI quality: results from the ESOR Nicholas Gourtsoyiannis teaching fellowship. Eur Radiol 33:461–471 35771247 10.1007/s00330-022-08947-5 PMC9244244

[32] Wang R, Pinto D, Liu T et al (2023) Effect of a dedicated PI-QUAL curriculum on the assessment of prostate MRI quality. Eur J Radiol 164:110865 37167684 10.1016/j.ejrad.2023.110865

[33] Belue MJ, Law YM, Marko J et al (2023) Deep learning-based interpretable AI for prostate T2W MRI quality evaluation. Acad Radiol 31:1429–1437 10.1016/j.acra.2023.09.030 PMC11015987 37858505

[34] Cipollari S, Guarrasi V, Pecoraro M et al (2022) Convolutional neural networks for automated classification of prostate multiparametric magnetic resonance imaging based on image quality. J Magn Reson Imaging 55:480–490 34374181 10.1002/jmri.27879 PMC9291235

